# Comparison of different estimators of SARS-CoV-2 pandemic activity on geographical and temporal levels

**DOI:** 10.3389/fpubh.2022.871010

**Published:** 2022-10-06

**Authors:** Remi Valter, Grace Sembajwe, Alexis Descatha, Marc Fadel

**Affiliations:** ^1^UFR Simone Veil, Univ Versailles Saint-Quentin-en-Yvelines, Montigny-le-Bretonneux, France; ^2^Department of Occupational Medicine, Epidemiology and Prevention, Hofstra/Northwell Health, New York, NY, United States; ^3^IRSET-ESTER UMR_S 1085, University and CHU of Angers, SFR ICAT, CAPTV CDC, Angers, France

**Keywords:** COVID-19, occupational health, public health, epidemiology, job-exposure matrices

## Abstract

**Background:**

Studies began investigating occupational exposures as a source of contamination to SARS-CoV-2, yet few considered the variation in SARS-Cov2 pandemic activity for these exposures. Several indicators were built to assess SARS-Cov2 activity though they usually serve a specific purpose and have limitations. The aim was to compare qualitatively different estimators of the SARS-CoV-2 pandemic activity and to create an estimator of pandemic activity level based on daily hospital admissions for job-exposure matrices (JEM) usage.

**Methods:**

From publicly available French databases, we retrieved all data from March 19, 2020 (first day available) to March 25, 2021 (day of data collection) on four different estimators: percentage of intensive care bed occupied, reproductive number, tests' positive rate and number positive tests. An indicator based on new daily hospital admissions was created for a COVID JEM. Due to the heterogeneity of the estimators, a qualitative comparison was carried out.

**Results:**

During the study period, three major outbreaks took place. Though the number of positive tests was the first indicator to worsen during the 2nd outbreak, it failed to identify variation during the outbreak. Though each indicators behaved differently during the study period, the indicator based on new daily hospital admissions and the positive rate seemed to be the closest to one another.

**Conclusion:**

This study highlights the heterogeneity of the indicators used during the first and second SARS-Cov2 outbreaks in France. An indicator based on new daily hospital admissions seems to be a good candidate for estimating SARS-CoV-2 epidemic activity for COVID JEMs and is easily available in countries where usual indicators are not commonly accessible.

## Introduction

While the COVID-19 crisis is still underway, questions have risen regarding occupational exposures as a source of contamination. Though temporarily shutting down work activities was a measure frequently used at the beginning of the pandemic, prolonged lockdowns seem unreasonable because of their adverse effect on the economy and on health (1, 2). Work life is now regimented by the variation in SARS-CoV-2 pandemic activity, which often changes how strict preventive measures would be applied. Thus, companies, with the help of occupational health professionals, are constantly having to adapt their work organization to the ebbs and flows of the SARS-CoV-2 infection trends. Indeed, assessing biological hazards, including infectious diseases, and implementing adequate preventive measures has become fundamental, and the International Labor Organization has recently released guidelines to advice governments, employers, workers and their representatives (3). There are several methodology to assess the workplace risks and management them consequently, but most of them characterize biological hazard in relation to the probability of contact to a contagious source, whether there are contacts with colleagues at work or the general public, and the means of protection including how likely a close proximity is needed at work (4). In the case of a infectious diseases, especially with airborne transmission, the first part of this assessment requires to know the epidemic activity level which can influence the probability of a contact to be a contagious source. However, this factor is often eluded even if there are exposure indicators that could be used to optimize employer and occupational health professionals' responses to pandemics, especially in the case of SARS-CoV-2.

Governments and research teams have built many models with exposure indicators to assess SARS-CoV-2 activity. Each indicator serves a specific purpose and is used in public health decision-making that is often guided by whether or not health systems are overloaded. One of the most common indicators used for assessing the SARS-CoV-2 epidemic activity is the daily number of positive PCR tests. These tests may seem like decent indicators of epidemic activity since they have good sensitivity and specificity (5) and permit early detection (6). However, they have many limitations, including the time needed to develop, validate and make them broadly accessible as well as their dependence on the number of tests performed (7). The daily number of new hospital admissions is an indicator closely related to the circulation of the virus (8). It is easily accessed and quickly useable. It seems like a reliable indicator in countries where surveillance capabilities are limited, beyond confirmed deaths from COVID-19. Cumulative incidence on a set period (weekly, monthly) also could be a better choice than daily indicators since the latter are more susceptible to variability and errors. However, considering the singularities of workplaces, the best usable indicator for occupational health practitioners and stakeholders is not known.

The aim of this study was to qualitatively compare different estimators of the SARS-CoV-2 epidemic activity that are currently in use as pandemic indicators, as well as create an estimator of pandemic activity that would be based on daily hospital admissions. These indicators were compared across both time and geographical variations.

## Method

### Origin of data

Data on COVID-19 indicators such as incident cases or incident new hospitalization cases were retrieved from France's official government website (9). Launched in May 2020, SI-DEP, a screening information system, is a secure platform where the results of SARS-CoV-2 tests from all hospitals, laboratories, pharmacies, healthcare professionals, and screening campaigns are systematically recorded. The SI-DEP information system feeds various institutions with different objectives and needs: Public Health France and the Ministry of Health for monitoring the epidemic; the France compulsory health insurance and regional health agencies for contact tracing. The database variables are publicly available and accessible at the departmental level (equivalent to county). We included all data from March 19, 2020 (the first day available) to March 25, 2021 (day of data collection). Since we aimed to build an indicator that would show the spatial trends of the SARS-Cov2 circulation on a county level, the indicator needed to be standardized on the number of inhabitants per county. For this, we retrieved data from the latest national census available (2020) (10).

### Estimators

Five different estimators were used in this study: four indicators provided by the French Government based on the daily cases of positive PCR, on the positivity rate of COVID tests, on the basic reproduction number and on the percentage of intensive care beds occupied), and a specific indicator made for Mat-O-Covid JEM based on the daily number of new hospital admission.

The first estimator is the number of people tested positive by a PCR or an antigen test for the first time in the last 60 days standardized by the number of inhabitants. The indicator is calculated for a moving week and categorized in three level of epidemic activity: <10 positive cases per 100.000 inhabitants (low epidemic activity), between 10 and 50 positives cases (moderate epidemic activity), ≥50 positive cases (high epidemic activity). Due to the lack of tests during the first covid outbreak, this indicator is usable only from May 13, 2020.

The second estimator used is the positive rate of COVID test which is the percentage of number of PCR or an antigen test positive divided by the number of tests carried out on a set period. Three level of epidemic activity were calculated: positive rate <5% (low epidemic activity), positive rate between 5 and 10% (moderate epidemic activity) and positive rate ≥10% (high epidemic activity). Positive rate was available from May 19, 2020.

The third estimator is the basic reproduction number which is calculated once a week based on data from the previous week. Three level of epidemic activity were calculated: basic reproduction number <1 (low epidemic activity), basic reproduction number between 1 and 1.5 (moderate epidemic activity), and basic reproduction number ≥1.5 (high epidemic activity). This estimator was available from June 15, 2020.

The fourth estimator is the percentage of intensive care beds occupied which is the number of patients hospitalized in intensive care unit divided by the number of ICB available before the COVID-19 crisis. Three level of were defined: percentage of ICB occupied <30% (low epidemic activity), percentage of ICB occupied between 30 and 60% (moderate epidemic activity), and percentage of ICB occupied ≥60 (high epidemic activity). This estimator was available from the beginning.

The last estimator used was built specifically for the Mat-O-Covid project, a COVID JEM (11). The estimator is based on the cumulated number of new hospital admissions on a weekly basis and is calculated for each county, from March 19, 2020 to March 25, 2021. This distribution of all cumulated number of new hospital admissions for each week and each county considered is saturated to lower the effect of extreme observations. The threshold used for this saturation was identified as the value equal to the third quartile plus 1.5 times the interquartile range. Using this saturated distribution, the maximum for the entire population was identified, and three categories of epidemic activity were created: ratio of incident number of new hospital admissions divided by the maximum number of new hospital admissions <1/3 (low epidemic activity, i.e., 12.0/100,000), between 1/3 and 2/3 (moderate epidemic activity) and >2/3 (high epidemic activity, i.e., 24.0/100,000).

Three of the four government estimators assess epidemic activity daily. To allow comparison with our estimator which was chosen to be weekly, we created a weekly average of epidemic activity for these estimators. The epidemic activity variable was converted into a discrete quantitative variable: low epidemic activity being “1,” moderate epidemic activity “2” and high epidemic activity “3.” The weekly epidemic activity corresponded to the rounded mean on a week.

For the analysis of these indicators, we decided to take a qualitative approach to illustrate our hypothesis, which is that classical indictors used in epidemy activity level are heterogeneous. Indeed, there is no gold standard to compare these indicators and each of them estimate different aspect of an epidemy activity which makes the comparison complex. As such, no quantitative estimates were made in this study. Three representative counties and one oversea county were chosen: Paris (most populated), Bas-Rhin (high epidemic activity during the first outbreak), Ille-et-Villaine (low epidemic activity during the first outbreak) and La Guadeloupe (overseas county). A table presentation showing evolution of all five estimator was created and a table with all counties is available as Supplementary material. All analyses were run using R software version 4.0.4 (packages “tidyverse” and “ggsci”). The new hospital admission indicator that was constructed is at an early stage of development and further work will be needed to better analyze its statistical and epidemiological attributes.

## Results

Between March 19, 2020 and March 25, 2021, data were collected for 371 days, i.e., 53 weeks. During this period, three major outbreaks took place: from March to April 2020 (first lockdown), from October to December 2020 (first curfew and second “soft lockdown”) and from March to April 2021 (extended curfew and third “soft” lockdown) (12). The only indicators available during the first lockdown were the percentage of ICB occupied and the new hospital admission.

**Figure 1:**
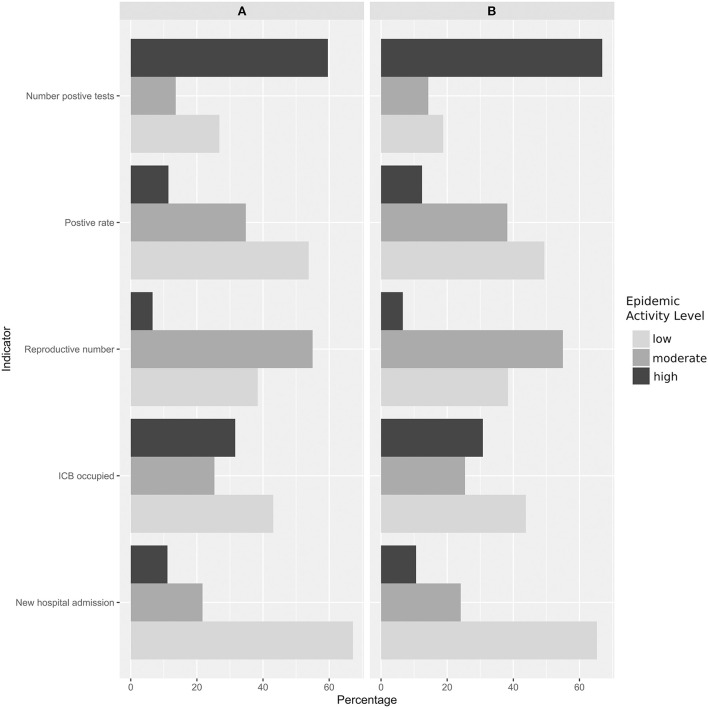
Percentage of weeks, for all counties, categorized as high, moderate, and low epidemic activity by the indicators considering **(A)** all the study period and **(B)** the date when all indicators were available (18 June 2020).

Though the number of positive tests was the first indicator to worsen during the 2nd outbreak, as early as September 2020, it classified weeks as high epidemic activity during the rest of the study period (until March 2021) for almost all counties (Table 1 and Supplementary Data). The percentage of intensive care beds occupied was the 2nd indicator that categorized the most weeks in high pandemic level activity, with more than 30% weeks classified as high epidemic activity (Figure 1). The reproduction number indicator classified the least weeks as high epidemic activity (6.4%) compared to the other indicator. Though each indicators behaved differently during the study period, the number of hospital admission indicator and the positive rate indicator seemed to be the closest to one another, though latest classified more weeks in moderate epidemic activity than low epidemic activity.

Table 1Comparison of the different indicators according to time for four different types of counties.

**Table:** 

	**Month**			**April 2020**				**May 2020**					**June 2020**				**July 2020**				**August 2020**				**September 2020**				
	**Week**	**0**	**1**	**2**	**3**	**4**	**5**	**6**	**7**	**8**	**9**	**10**	**11**	**12**	**13**	**14**	**15**	**16**	**17**	**18**	**19**	**20**	**21**	**22**	**23**	**24**	**25**	**26**	**27**
**Counties**	**Indicator**																												
Paris	New hospital admission																												
Paris	Intensive care bed occupied																												
Paris	Reproductive number																												
Paris	Positive rate																												
Paris	Number positive tests																												
Bas-Rhin	New hospital admission																												
Bas-Rhin	Intensive care bed occupied																												
Bas-Rhin	Reproductive number																												
Bas-Rhin	Positive rate																												
Bas-Rhin	Number positive tests																												
Ille-et-Villaine	New hospital admission																												
Ille-et-Villaine	Intensive care bed occupied																												
Ille-et-Villaine	Reproductive number																												
Ille-et-Villaine	Positive rate																												
Ille-et-Villaine	Number positive tests																												
La Guadeloupe	New hospital admission																												
La Guadeloupe	Intensive care bed occupied																												
La Guadeloupe	Reproductive number																												
La Guadeloupe	Positive rate																												
La Guadeloupe	Number positive tests

**Table:** 

	**Month**	**October 2020**				**November 2020**					**December 2020**				**January 2021**				**February 2021**				**March 2021**				
	**Week**	**28**	**29**	**30**	**31**	**32**	**33**	**34**	**35**	**36**	**37**	**38**	**39**	**40**	**41**	**42**	**43**	**44**	**45**	**46**	**47**	**48**	**49**	**50**	**51**	**52**	**53**
**Counties**	Indicator^*^																										
Paris	New hospital admission																										
Paris	Intensive care bed occupied																										
Paris	Reproductive number																										
Paris	Positive rate																										
Paris	Number positive tests																										
Bas-Rhin	New hospital admission																										
Bas-Rhin	Intensive care bed occupied																										
Bas-Rhin	Reproductive number																										
Bas-Rhin	Positive rate																										
Bas-Rhin	Number positive tests																										
Ille-et-Villaine	New hospital admission																										
Ille-et-Villaine	Intensive care bed occupied																										
Ille-et-Villaine	Reproductive number																										
Ille-et-Villaine	Positive rate																										
Ille-et-Villaine	Number positive tests																										
La Guadeloupe	New hospital admission																										
La Guadeloupe	Intensive care bed occupied																										
La Guadeloupe	Reproductive number																										
La Guadeloupe	Positive rate																										
La Guadeloupe	Number positive tests																										

* Black square = high pandemic activity, dark gray square = moderate pandemic activity, light gray square = low pandemic activity.

## Discussion

This study highlights the heterogeneity of the indicators used during the first and second SARS-CoV-2 outbreaks in France.

There is no gold standard for assessing the epidemic level activity of a disease and finding a good indicator can be difficult. The European Center for Disease Prevention and Control (CDC) enumerates a number of qualities a good indicator should have (13). Some qualities are related to the inherent qualities of the indicator, such as its ability to measure adequately (e.g., sensitivity, reliability), others are focused on the usage of the indicator, such as, its simplicity or its representativeness. The World Health Organization further develops these characteristics: an indicator should be relevant, scientifically sound and applicable to users (14). Though it seemed intuitive to use and easy to understand by everyone, the number of positive test as an indicator of epidemic activity did not seem to be sensitive to changes. Likely, the reproductive number worsened early during the 2nd outbreak but tended to underestimate peaks of epidemic activity.

The indicator based on the number of new hospital admission cases seems to be a good candidate for estimating SARS-CoV-2 epidemic activity. First, new hospital admission is a variable that can be easily obtained even at the beginning of a pandemic and in countries where PCR tests are not easily available since hospitalization data is now always collected. Second, it is also a simple and understandable indicator to use as it is not conceptual (number of cases) and as it also reflects the burden of SARS-CoV-2 on health systems. Lastly it is also more robust to differential bias as the criteria for hospital admission is consistent to both time and geographical areas, contrary to the number of positive tests for example, which is dependent on the number of tests performed and its availability. The main limitation is that it seemed to underestimate epidemic activity in counties less populated (Supplementary material) and worsened later than some indicators. This may be due to how the indicator was constructed as the thresholds used are based on the total number of cases by week, which is a national data, and thus flatten the epidemic activity assessment in these counties. This potential new indicator is still in development and much research will be needed, notably to assess its statistical and epidemiological proprieties before considering a potential usage as indicator of epidemic activity.

As mentioned previously, management of occupational biological risk hazard is essential for preventing propagation of diseases. This assessment will allow to implement preventive measures that are proportionate to the intensity of the workplace risk and clinical vulnerability risk (4). Indeed, prevention measures that are too strict can lead to adverse health effect as shown by the SARS-Cov2 pandemic (2). A precise assessment of the risks is thus important and could need indicators of level of epidemic activity. This work suggest that some indicators are better suited for this assessment, and the reproductive number and new hospital admissions indicators could be used on a county level to help workplace adapt their prevention measures. For example, occupational health professionals could first assess the risk of contact with public and colleagues during their work. If this risk is high, they could next use local and open access data from public health agencies (like new hospital admission or positive rate) to incentivize broader teleworking or social distancing at work when these indicators begin to worsen or increase past a threshold. Other potential targets for mitigating risk could be promoting more frequent testing, enabling contact tracing, and incentivizing vaccination, if tests and vaccine are available. Likely, a decrease of these indicators would help alleviate preventive measure. This kind of approach would allow a flexibility in the implementation of safety measures and would also consider both the local trend of pandemics and the specificity of workplaces.

In addition, on a broader level, new hospital admission could be an interesting indicator to use for Covid-19 job-exposure matrices (JEM) for research and public health purposes. For example, The Mat-O-Covid project (“Matrix-Occupation-Covid”) aims to build a job-exposure matrix (JEM) for SARS-CoV-2 exposure. JEM allow to have a mean estimate of exposure according to a job title. JEM have many strengths and weaknesses (15) and, while not being a good estimate on an individual level, the results of JEM are useful when working on a population level. While this indicator was developed for the French JEM Mat-O-Covid, it could be adapted for other covid JEM that are being constructed to further improve their estimations (16). Epidemic activity is an important factor to consider in these matrices due its variability according to time and geographic area, as illustrated in this study.

The descriptive analysis limits the results of this study, however, and a direct comparison between the indicators would not be relevant due to the difference in what they measure. The lack of gold standard also makes it difficult to validate the indicators. In many countries, the problem is about the availability of such indicators, and the indicator based on new hospital admission seems promising, though much statistical confirmation is needed before implementing it. Our work illustrates some strengths and limitations of each indicator though careful interpretation is warranted as they are not easily interchangeable and assessing the level of epidemic activity would require using more than one to be thorough.

To conclude, this study highlights the heterogeneity of the indicators used to assess SARS-CoV-2 epidemic activity. An indicator based on new hospital admission may be useful for workplace decision-making, future COVID JEM and in countries where usual indicators are not commonly accessible.

## Data availability statement

The raw data supporting the conclusions of this article will be made available by the authors, without undue reservation.

## Ethics statement

Ethical review and approval was not required for the study on human participants in accordance with the local legislation and institutional requirements. Written informed consent for participation was not required for this study in accordance with the national legislation and the institutional requirements.

## Author contributions

RV, AD, and MF designed the study. RV and MF collected the data, carried out data analysis, and drafted the manuscript. GS and AD critically reviewed the manuscript. All authors contributed to the article and approved the submitted version.

## Funding

This study was supported by REACTing Inserm (Mat-O-Covid project), ANRS Maladies infectieuses émergentes since 2021), and Regional public fund (TEC-TOP project, Pays-de-la-Loire Region, Angers Loire Métropole, Univ Angers/CHU Angers).

## Conflict of interest

Author AD is editor in chief of Archives des maladies professionnelles et de l'environnement (Elsevier).

The remaining authors declare that the research was conducted in the absence of any commercial or financial relationships that could be construed as a potential conflict of interest.

## Publisher's note

All claims expressed in this article are solely those of the authors and do not necessarily represent those of their affiliated organizations, or those of the publisher, the editors and the reviewers. Any product that may be evaluated in this article, or claim that may be made by its manufacturer, is not guaranteed or endorsed by the publisher.
